# Tobacco Use and Smoking Cessation Practices among Physicians in Developing Countries: A Literature Review (1987–2010)

**DOI:** 10.3390/ijerph110100429

**Published:** 2013-12-30

**Authors:** Abu S. Abdullah, Frances A. Stillman, Li Yang, Hongye Luo, Zhiyong Zhang, Jonathan M. Samet

**Affiliations:** 1 School of Public Health, Guangxi Medical University, Nanning 530021, China; E-Mails: yangli8290@hotmail.com (L.Y.); Hongye283@163.com (H.L.); rpazz@163.com (Z.Z.);; 2 Department of Medicine, Boston University School of Medicine and Boston Medical Center, Boston, MA 02218, USA; 3 Johns Hopkins Bloomberg School of Public Health, Baltimore, MD 21205, USA; E-Mail: fstillma@jhsph.edu; 4 Department of Preventive Medicine, Keck School of Medicine of USC, USC Institute for Global Health, University of Southern California, Los Angeles, CA 90033, USA; E-Mail: jsamet@med.usc.edu

**Keywords:** tobacco use, physicians, developing countries, smoking cessation, review literature

## Abstract

Physicians have a key role to play in combating tobacco use and reducing the tobacco induced harm to health. However, there is a paucity of information about tobacco-use and cessation among physicians in developing countries. To assess the need for and nature of smoking cessation services among physicians in developing countries, a detailed literature review of studies published in English, between 1987 and 2010 was carried out. The electronic databases Medline and Pub Med were searched for published studies**.** The findings show that there are regional variations in the current smoking prevalence, quitting intentions, and cessation services among physicians. Smoking prevalence (median) was highest in Central/Eastern Europe (37%), followed by Africa (29%), Central and South America (25%) and Asia (17.5%). There were significant gender differences in smoking prevalence across studies, with higher prevalence among males than females. Smoking at work or in front of patients was commonly practiced by physicians in some countries. Asking about smoking status or advising patients to quit smoking was not common practice among the physicians, especially among smoker physicians. Organized smoking cessation programs for physicians did not exist in all of these regions. This review suggests that while smoking of physicians varies across different developing regions; prevalence rates tend to be higher than among physicians in developed countries. Quitting rates were low among the physicians, and the delivery of advice on quitting smoking was not common across the studies. To promote tobacco control and increase cessation in populations, there is a need to build physicians’ capacity so that they can engage in tobacco use prevention and cessation activities.

## 1. Introduction

Tobacco smoking is the leading cause of preventable death world-wide. Currently, tobacco is responsible for 5 million deaths annually. This annual toll is expected to increase to 10 million within the next 20–30 years, with 70% of deaths occurring in developing countries (World Health Organization (WHO)) [1]. Discouraging initiation and reducing the use of tobacco is currently among the most important public health strategies that countries can implement. Because of their close contact with the public, and the fact that physicians are role models, opinion leaders and often decision makers for healthcare policy, physicians can play a key role in efforts to reduce smoking. Research has shown that smoking cessation interventions by physicians are both efficacious and cost-effective with regard to patient smoking outcomes [2]. Smoking cessation in large number of smokers, as could be delivered by physicians, could reduce the epidemic of tobacco deaths in the next few decades [3]. 

In developed countries physicians play a key role in promoting smoking cessation [4], and recommendations to engage physicians more actively in the smoking cessation effort go back decades [5]. In developing countries, however, physicians are less involved in tobacco control and smoking cessation efforts [6], perhaps because a substantial proportion of physicians in these countries are smokers themselves. In a recent review, Abdullah *et al*. reported that smoking prevalence among Chinese male physicians ranged from 26% to 61% [7]. A high smoking prevalence was also reported among physicians in several other developing countries. For example, the prevalence was as high as 40% [8] among Bosnian and 50% [9] among Pakistani male physicians. During the last two decades, a number of studies have addressed tobacco use among physicians in developing countries, but many have been published in local journals and were not accessible internationally. At the same time, there have been significant changes in the tobacco control policies across the nations during the last decade. An understanding of the differences in the findings from earlier studies to that of newer studies would provide insights about the changing trajectories of tobacco use by and cessation counseling delivered by physicians in developing countries.

To provide a summary of research on tobacco use and cessation counseling practices involving physicians in developing countries, we reviewed literatures published during the last three decades. 

## 2. Methods

A literature review of all articles published in peer-reviewed journals that included data on prevalence of physician smoking in developing countries was conducted. Developing countries (low and middle income economies), as defined by the World Bank [10], were grouped under four regions: Africa, Asia, South/Central America and Central/Eastern Europe. A significant amount of literature was available from developed nations but was not included in this review because of the focus on developing countries. The literature review began with searches on Medline and PubMed using four combinations of medical subject headings (MeSH): *physician* (including subheadings: physician’s role; physician’s practice patterns; and “physicians” as a keyword) and *tobacco* (including: tobacco, smokeless; tobacco use cessation; and “tobacco” as a keyword); *physician* and *smoking* (including: smoking cessation and “smoking” as a keyword); “doctor” (as keyword only) and *smoking*; and “doctor” and *tobacco*. We restricted our review to materials published in the last 33 years (1987–2010), to provide a review since the tobacco use related information was available in the literature. 

All English language articles for which a full-text version could be found were included in the review if they reported prevalence of smoking among physicians or health care workers. Abstracts for publications for which full-text versions were unavailable were also combed for prevalence data. For foreign language articles, an attempt was made to acquire English versions by contacting the corresponding authors. If no response was forthcoming, we similarly reported the prevalence data from the available English abstract. Where no English abstract was available, we excluded the article after an attempt to contact the corresponding author. We also scanned the references of articles reviewed for additional materials, but this did not yield many articles that met the criteria of our study. Manuscripts were arranged by region and in descending order of year of publication. We used publication date rather than study date for consistency, since publication dates were always available and study dates were not. Although a thorough review of grey literature was not conducted, we did use the WHO country reports providing prevalence data on physician smoking. Studies that were conducted among medical students were not included. Also studies that were conducted among healthcare workers but did not include physicians were excluded.

We assigned each manuscript a reference number based on the criteria mentioned above. We included the response rate for the studies where available and rounded this to the nearest whole number. We also provided sample size, indicating the total number of respondents (not the number originally surveyed). We provided smoking prevalence, with gender breakdown where available, and also information about the number of cigarettes smoked per day. Where physician smoking prevalence was not reported separately from other health workers, we reported the combined figure. We also noted other key findings as appropriate.

## 3. Results

### 3.1. General Descriptions

We identified 59 full-text published studies that fit our criteria. Only one study [11] addressed more than one country in the same paper. We reviewed 10 additional abstracts where full-text English articles were not available. We also included the data from 10 countries assessed in the WHO’s Tobacco or Health Report, and 13 WHO country fact sheets. In total, we had 92 separate sources of data on smoking among physicians. The regional breakdown for all sources was as follows: nine from Africa, 28 from Asia, 31 from Central/Eastern Europe, and 24 from Central/South America. Sample sizes ranged from as high as 20,848 [12] to as low as 63 [13] across studies. As shown in Table 1, most of these studies were cross-sectional in nature and used postal or self-administered questionnaire surveys to collect data. Only one study, in Russia, conducted in-person interviews [14]. For twelve sources, methods of data collection were unreported or unclear. 

### 3.2. Smoking among Physicians

#### 3.2.1. Prevalence of Tobacco Use

Overall smoking prevalence rates across studies ranged from as high as 63% in the Philippines [1] and 62% in Mexico [15], to as low as 3% in Nigeria [16]. In eight studies, the overall smoking prevalence rate was under 10% and in four studies it was 50% or above. 

#### 3.2.2. Gender Difference

In fifty seven studies that reported gender-specific tobacco use prevalence, smoking prevalence was higher among male than female physicians. In addition, at least in Europe, male smokers smoked more cigarettes per day than their female counterparts [8,17]. Among both African and Asian physicians, the gender difference was especially pronounced, with most studies reporting the smoking prevalence rate among females as below 5%, while males smoked as much or more than males in other regions. In Eastern and Central Europe, females smoked notably less than males in most of the studies. However, in a 1996 study, the daily smoking rate was higher among Bosnian female physicians (55%) than their male counterparts (50%) [18]. In Central/South America, the female rates, although still lower, track more closely with male rates. In a Mexican study, females actually smoked more than males: 39% *vs.* 22% [19]. 

#### 3.2.3. Age at First Smoking

Eleven studies reporting the age of onset found the mean or the mode to be under the age of 20 [11,20,21,22,23,24,25]. In general, males started smoking earlier than females, usually before the age of 18 [6,22,26,27]. 

#### 3.2.4. Number of Cigarettes Smoked per Day

Twenty articles reported the number of cigarettes smoked per day. However, methods varied: some reported average daily cigarette consumption while others reported a range (e.g., <10 cigarettes per day, 10–20 cigarettes per day, *etc.*). Others studies categorized smokers as light, moderate or heavy, with varying definitions, making comparisons among studies somewhat difficult. Some trends do emerge, however, from the data, especially in regional comparisons.

**Table 1:** Description of Tobacco use and cessation related surveys among physicians between 1986 and 2010 by regions.

Author [ref.]	Country	Year of Publication	Study Population	Sample Size	Study Design	Response Rate	Def of Smoker	Total Prev. [avg # of cig/day]	Male Prev.	Female Prev.	Other Relevant Findings and Recommendations
**Africa Region**											
Nollen, *et al.* [16]	Nigeria	2004	Physicians in two teaching hospitals	373	Cross-sectional survey	80%	-	3%	-	-	Suggested to implement smoking bans in health facilities.
Tessier, *et al.* ^#^ [11]	Algeria	1999	Health Professionals	-	Secondary data analyses	-	-	40%	-	-	
	Morocco	1999	Health Professionals	-	Secondary: data analyses	-	-	30%	-	-	
	Egypt	1999	Health Professionals	-	Secondary data	-	-	40%	-	-	
Ndayie, *et al.* * [28]	Senegal	1999	Physicians	163	Cross-sectional study	-	Current smoker	28%	-	-	More male than female smokers. 56.4% were heavy smokers. 70% of physicians smoked at work.
											Recommended to create special smoking cessation programs targeted at doctors.
Rady and Sabbour [15]	Egypt	1997	Physicians at Ain Shams University	382	Behavioral risk factor surveillance system	62%	Current smoker	-	27%	3%	51% of current smokers reported smoking in front of patients or in clinic. 2/3 of current smokers have tried to quit smoking during the past year. Smoking is more prevalent among young physicians.
Shafey, *et al.*, (Eds.) [18]	Egypt	1993	Physicians	-	-	-	-	34%	-	-	
WHO (1997) [1]	Mada-gascar	1993	Physicians	-	Survey	-	Current smoker	29%	-	-	Surgeons had higher rate of smoking (44%)
Callander and Rocke [29]	South Africa	1986	Anesthetists in Durban	102	Survey	78 %	Daily smoker	19%	-	-	80% of smokers had considered quitting. Most highly rated reason for not smoking was health protection. There was a significant difference between smokers/non-smokers regarding perception of smoking as a health hazard.
											Suggested for physicians support on policy development, bans on smoking in health facilities, and health info on cigarette packets.
Ballal [30]	Sudan	1984	Medical Practitioners	753	Crosssectional survey	72.4 %	Current smoker	-	46%	<0.1%	More than half of smokers smoked 15 cigs/day or greater. Subjects who engage in heavy smoking (>14 cig/day) have more difficulty in quitting.
Shafey, *et al.* (Eds.) [18]	Ethiopia	1983	Health Professionals	-	-	-	-	9%	13%	2%	
Asia Region											
Vanphanom, *et al.* [31]	Laos	2011	physicians	855	Survey	80.6	Daily smoker	9.2%	17%	0.4%	Older doctors smoked more than the younger doctors. Over 84% of current smokers wanted to quit, and 74.7% had made a recent serious attempt to do so. Only 24% had received cessation training; 8.8% considered themselves ‘well prepared to deliver counseling
Zhou, *et al.* [6]	China	2010	Physicians	673	Survey	85%	Current smoker	26%	35%	3%	50% reported that no smoke-free policy was in place in their hospital;93% had not received any training on smoking cessation counseling, and 62% had not read any smoking cessation guidelines.
Malik, *et al.* [9]	Pakistan	2010	Physician	234	Survey	88%	Current smoker	37.2%	50.3%	7%	Doctors considered smoking as relaxing; Peer pressure was a triggering factor for smoking
Peykari, *et al.* [32]	Iran	2010	general practitioners	5,140	Cross sectional	-	Life time current	15% (6.6 cig/d)	-	-	There was significant relationship between smoking pattern of GPs and their knowledge about harms from smoking, and attitude towards smoking.
Lam, *et al.* [33]	China	2010	Male physicians	514	Cross sectional	60.8%	Current smoker	-	24.9%	-	Non-smokers than smokers were more likely to advice on quitting. Factors significantly associated with ‘‘initiation and/or advice’’ were prior smoking cessation training, a non-smoking status, better knowledge of smoking cessation and organizational support.
Shi, *et al.* [34]	China	2010	Anesthesiologists	467	survey	60.3%	Current smoker	10%	18.4%	3.9%	Compared with nonsmokers, smokers were significantly less likely to advise about the health risks of smoking and quitting.
Ceraso, *et al.* [35]	China	2009	Male physicians	103	survey	89%	Current smoking (Smoking in the last month or 100 cigarettes life time smoking)	49.5%	49.5%	-	More than one-fourth (29%) of non-smoking physicians accepted cigarettes as gifts, and these physicians were less likely to ask their patients about their smoking status than those who did not accept gift cigarettes. Only 19% reported receiving training in how to help their patients quit.
Fadhil., L., *et al.* [36]	Bahrain	2009	Primary care physicians	120	survey	55%	Current smoker	24%	-	-	Only 4% physicians received training about tobacco cessation interventions.
Han Zao, L., *et al.* [37]	China	2008	physicians	347	survey	86.8%	Current smoker	42%	58%	18%	The following five variables were significantly associated with physicians’ smoking cessation counseling frequency: their smoking status, perceived success in their past counseling, perceived influence,perceived exemplary role, and perceived responsibility
Merrill, R., *et al.* [26]	Jordan	2008	Physicians and nurses	513(physician = 251)	Cross sectional	-	Daily smoker	12% (16/d)	-	-	Besides daily smokers, 7% were occasional smoker and 17% were former smoker; 81% of physicians who currently or formerly smoked had smoked in front of patients. Smoking status and training were associated with counseling patients about smoking.
Yan, J., *et al.* [38]	China	2007	Physicians and nurses	636 (physician = 358)	Cross sectional	77.56%	Current Smoker	20.8% (16/d)	43%	1.2%	45% of physicians informed patients about methods of smoking cessation. Smoking behavior was negatively associated with giving cessation counseling.
Ng, N., *et al.* [39]	Indonesia	2007	Physicians	447	Cross sectional	65%	Current smoker	-	22%	1%	72% of physicians did not routinely ask about their patient’s smoking status.
Jiang *et al* [40]	China	2007	Hospital based physicians	3,552	Clustered randomized survey	-	Current smoker	23% (less than a pack)	41	1	Prevalence of current smoking increased with age except in physicians aged >60; Former smoking rate 2.7% (male 4.7%, female 0.3%)
Smith, *et al.* [41]	China	2006	Physicians in Hebei Province	361	Self-reporting survey	79%	Current smoker	16 % (10 c.)	32% (10 c.)	0%	51.4% were light smokers; only 9% heavy smokers.
Mohan, *et al.* [20]	India	2006	Physicians	229	Cross sectional survey	86%	Current smoker	16%	13%	0%	About 60% had made some effort to quit in last year (1/3 had tried 4+ times). Recommended inclusion of tobacco control education in medical curriculum with a focus on dangers of low-level smoking.
Shafey, *et al.* (Eds.) [18]	Palau	2001	Health professionals (MOH)	-	Survey	-	Current smoker	-	20 %	15 %	
Li, *et al.* [42]	China	1999	Physicians	493	Survey	82%	Current smoker	-	61%	12%	One third smoked 20 or more cigs per day. Argued that physicians should be educated about their role as a role model in the society.
Shafey, *et al.* (Eds.) [18]	Laos	1996	Physicians	-	Survey	-	Current Smoker	18%	-	-	
Shafey, *et al.* (Eds.) [18]	Tonga	1994	Physicians	-	Questionnaire survey	-	Current smoker	-	14%	0%	
Yaacob, *et al.* [43]	Malaysia	1993	Physicians at a university hospital	120	Mailed survey	-	Current smoker	18%	25%	0%	2/3 of smokers had previously tried to quit. Majority of smokers smoked 11–20 cigs/day.
WHO (1997) [1]	Fiji	1991	Physicians	-	Cross-sectional survey	-	Regular smoker	26%	-	-	
	Mongolia	1991	Doctors at University Hospital	-	-	-	Current smoker	12%	-	-	
	Papua New Guinea	1990	Physicians	-	-	-	Current smoker	-	2%	-	
Sarkar, *et al.* [44]	India	1990	Physicians and medical students	218	Cross-sectional survey	98%	Current smoker	32%	48%	3%	About 1/3 each of heavy, moderate, and light smokers. 2/3 made efforts to quit and 9/10 had considered quitting. Physicians who smoked counseled patients significantly less often than non-smokers.
WHO (1997) [1]	Philippines	1987	Physicians	-	Survey	-	-	-	63%	37%	38% of all said they smoked in front of patients. Only 59% advised patients on harmful effects of smoking.
	Thailand	1987	MOPH doctors and dentists	-	-	-	-	17%(12.6 c.)	-	-	
	Mongolia	1980	Military doctors	-	-	-	Current smoker	50%	-	-	
Shafey, *et al.* (Eds.) [18]	Vietnam	-	Health workers	-	Cross-sectional survey	-	Tobacco product use	-	42%	1%	
Supramaniam [45]	Malaysia	1980	Military physicians	103	Postal questionnaire survey	87%	Current smokers	-	50%	-	Half are heavy smokers (smoked 20 or more cig/day). Only 20% are light smokers (less than 10/day).
East and Central Europe											
Perrin, *et al.* [46]	Armenia	2006	Physicians in Yerevan	240	Self-administered anonymous survey	70 %	Regular or occasional smoker	-	55%	17%	Male physicians started smoking at much younger ages (most <18) than women (most >25). 13% had smoked in front of patients. About 1/2 of men and 3/4 of women ready to quit now or in next 6 months. Smokers were less likely to counsel patients to quit.
Schnoll, *et al.* [13]	Russia	2006	Oncologists	63	Brief survey	-	Current smoker	27%	-	-	Almost 1/2 of physicians do not feel confident in providing counseling and more than 1/2 do not see counseling as being ineffective.
Squier, *et al.* [27]	Ukraine	2006	General practitioners	799	Cross-sectional survey	-	Current smoker	14%	62% (ever smoked)	21% (ever smoked)	Most smokers are light smokers (66%, less than 1/2 pack per day). Suggested to increase number of smoke-free places, starting in hospitals and healthcare facilities.
Poanta, *et al.* [47].	Romania	2006	Physicians in Cluj County	112	Cross-sectional survey	95%	Tobacco use	42%	55%	35%	
Parna, *et al.* [48]	Estonia	2005	Physicians	2,746	Postal Survey	68 %	Current smoker	-	25%	11%	Over half of the physicians tried to quit smoking. Physicians should be informed of the importance of their ability to be role models as non-smokers
Gunes, *et al.* [49]	Turkey	2005	Physicians at Turgut Ozal Medical Center	257	Cross-sectional survey	85 %	Current smoker	38%	-	-	
Hodgetts, *et al.* [9]	Bosnia and Herze-govina	2004	Physicians in 19 Family Medicine Teaching Centers	112	Cross-sectional survey	73%	Current smoker	40%(16 c.) * both nurses and phys.	-(2 cig) * both nurses and phys	40%(15 c.) both nurses and phys	Population overwhelmingly (91%) female. Those who had tried to quit in the past smoked significantly less cigarettes per day (14 *vs.* 22). Rates for male physicians not given due to small numbers.
Arkvadar, *et al.* [50]	Turkey	2004	Physicians	153	Cross-sectional survey	51 %	Current smoker	38%(12 c.)	-	-	
Zolnierczuk-Kieliszek, *et al.* [51]	Poland	2004	Hospital Staff	127	Cross-sectional survey	-	Current smoking	29%	-	-	57.4% of smokers tried to cut down while on hospital premises.
Glavas, *et al.* [52]	Croatia	2003	Health Professionals	119		97%	Daily smoker	37%	37%(both phys and nurses)	36%(both phys and nurses)	Gender specific prevalence were combined for physicians and nurses. 48.2% smoked 16–25 cigarettes daily. 67.5% have a strong desire to quit; 52 % had tried to quit.
Didilescu and Muntueanu [53]	Romania	2000	Physicians	1,136	-	-	-	43%	50%	39%	More than half of smokers smoke between 10–20 cig/day.
Shafey, *et al.*, (Eds.) [18]	Georgia	1998	Health Professionals	-	-	-	Current smoker	-	48%	16%	
	Russia	1999	Physicians in Moscow (30-70 years)	-	-	-	Current smoker	27%	41%	13%	
Wold, *et al.* [54]	Georgia	1999	Physicians and nurses	107	Cross-sectional survey	-	Tobacco product use	32%	-		
Shafey, *et al.*, (Eds.) [18]	Czech Republic	1998	Physicians	-	Cross-sectional survey		Daily or occasional smoker	-	26%	21%	
Shafey, *et al.*, (Eds.) [18]	Moldova	1998	Physicians	-	Cross-sectional survey	-	Current smoker	-	44%	6%	
	Ukraine	1998	Health Professionals	-	Secondary data	-	-	-	43%	19%	
	Bosnia	1996	Physicians	-	Survey	-	Regular daily smoker	-	50%	55%	
	Bulgaria	1996	Doctors	-	Survey.	-	Regular daily smoker	52%	-	-	
	Slovenia	1996	Physicians	-	Survey	-	Regular daily smoker	-	17%	15%	
	Poland	1995	Physicians	-	Cross-sectional survey	-	Regular daily smoker	-	24%	16%	
Kralikova, *et al.* [55].	Czech Republic	1995	Physicians	2,046	Survey	-	Current or occasional smoker	-	38%	26%	Almost half of physicians had not heard of NRT. 75% smoking physicians would like to stop smoking.
Shafey, *et al.*, (Eds.) [18]	Croatia	1993	Health Workers	-	Survey	-		35%	-	-	
	Latvia	1993	Physicians	-	-	-	Current smoker	59%	-	-	
	Turkey	1993	Physicians	-	-	-	Daily smoker	-	53%	41%	
	Lithuania	1992	Physicians	-	Survey	-	Current smoker	-	38%	10%	
Icli, *et al.* [56]	Turkey	1992	Residents and interns	200	Survey	100%	Current smoker	34%	35%	31%	About 62% had unsuccessfully tried to quit. Smoking residents are more likely to believe their influence on patient smoking is only minor, and less likely to offer counseling.
Gorecka, *et al.* [57] *	Poland	1991	Pneumol-ogists	850	Self-reported questionnaire survey	-	-	-	38%	29%	
Fortic [58] *	Slovenia	1989	Physicians	-	-	-	Regular smoker	-	30%	20%	Recommended that tobacco control and smoking cessation teaching should be mandatory at the medical schools.
Misiavichene, *et al.* [14]*	Russia	1987	Physicians	275	Interview	-	Regular smoker	-	30%	2%	Recommended to focus preventative measures on men, who have high rates of smoking.
Innos, *et al.* [17]	Estonia	1982(pub 2002)	Physicians	3,791	Survey	81%	Current smoker	21%	41%	15%	About 40% (60% of men, 1/4 of women) smoked 10 or more cigs/day
**Central/South America**											
Mejia, R., *et al.* [59]	Argentina	2010	Gynecologist	235	Survey	78.3	Current smoking	35%	-	-	Only 22% had received training in smoking cessation counseling and 48.5% reported insufficient knowledge to provide smoking cessation advice.
Viegas, *et. al.* [21]	Brazil	2007	Physicians, Federal District	830	Mailed surveys	12%	Regular or occasional smoker	7%	9%	5%	53% of smokers smoked 10 cig or less per day. 77% of smokers believed they could quit, and 68% intended to quit.
Balbani, *et al.* [22]	Brazil	2006	Otorhino-laryngologists	209	Mailed surveys	35%	Regular or occasional smoker	7%(14 c.)	-	-	73.3% of smokers have already tried to quit smoking.
Varona, *et. al.* [60]	Cuba	2005	Family physicians	121	Cross-sectional survey	-	Current smoker	18%	21%	16%	
Bello, *et al.* * [12]	Chile	2004	Employees of Chilean MOH	20,848	Cross-sectional survey	-	Current smoker	41%(7 c.)	43%	40%	45 % of smokers are interested in quitting.
Sanchez and Lisanti [23] *	Ecuador	2003	Physicians in Azuay, Ecuador	687	Survey	78%	-	32%	-	-	Men smoked more cigs per day than women.
Barnoya and Glantz [61]	Guatemala	2002	Physicians	174	Self-administered questionnaire survey	64%	Current smoker	18%(2.5 c.)	26%	5%	40% of residents who smoked said they did so at work. 76% of smokers said they would like to quit.
Salmeron-Castro [62] *	Mexico	2002	Physicians at the Mexican Institute of Social Security	3,133 (all workers)	Self-administered questionnaire survey	-	Current smoker	-	21%	16%	
Shafey, *et al.*, (Eds.) [18]	Uruguay	2001	Medical doctors	-	National survey	-	Current smoker	27%	-	-	
Grossman, *et al.* [63]	Costa Rica	1999	Physicians	217	Cross-sectional survey	76%	Current smoker	19%(<10 cigs)	59% (ever smoker)	60% (ever smoker)	2/3 of physicians had smoked in their office.
Sansores, *et al.* [19]	Mexico	1999	Physicians at National Institute of Health	4,422 (all health workers)	Survey	31%	Current smoker	22%	22%(all health workers)	39%(all health workers)	29% of physician smokers want to quit and 44% have quit at some point in the past. Place more restrictions on smoking in the workplace (or enforce current ones). Provide smokers who want to quit with effective health promotion and health education programs.
Tapier-Conyer, *et al.* [24]	Mexico	1997	Physicians	3,488	Survey	98%	Current smoker	27%	30%	21%	60% of men and 77% of women smoked≤10 cigarettes/day. Recommended to ban smoking in workplace to force quitting behavior or at least reduce number smoked during office hours.
Shafey, *et al.* (Eds.) [18]	Argentina	1997	Physicians at 15 Hospitals in Buenos Aires	-	Cross-sectional survey	-	Current smoker	30%	-	-	
Mirra and Rosemberg [25] *	Brazil	1997	Physicians	11,909	Cross-sectional survey	-	Regular smoker	6%	-	-	Onset of smoking most frequently between 10 and 19 years.
Shafey, *et al.*, (Eds.) [18]	Cuba	1995	Physicians	-	Cross-sectional survey	-	Daily smoker	25%	-	-	
	Panama	1993	Health Professionals (30–44 years)	-	Cross-sectional survey	-	-	10%	16%	5%	
	Peru	1993	Physicians (25 plus)	-	-	-	Current smoker	26%	27%	19%	
Cornejo, *et al.* [64] *	Chile	1992	Physicians	288	Questionnaire survey	-	Daily smoker	35%(9 Cig)	40%	24%	50% of smokers would not not quit smoking.
Shafey, *et al.*, (Eds.) [18]	Colombia	1991	-	-	-	-	Daily smoker	21%	21%	22%	
Ramirez-Casanova, *et al.* [65] *	Mexico	1991	Physicians	284	Questionnaire survey	-	Current or former smoker	61%	67% (among all health workers)	58% [amg all health workers]	Physician rates are comparable to overall prevalence in hospital workers. Implement a smoke free policy at the hospital.
Shafey, *et al.*, (Eds.) [18]	Paraguay	1989	Physicians (Age 20–80)	837	-	-	Current smoker	32%	35%	33%	
Alonso and Diaz [66] *	Chile	1989	Physicians from Valparaiso area	174	Survey	-	Current smoker	25%	-	-	
Shafey, *et al.*, (Eds.) [18]	Bolivia	1987	Physicians	-	Survey	-	Daily smoker	35%	-	-	
	Dominican Republic	1986	Physicians	-	-	-	Current smoker	-	43%	17%	

* Indicates that only the abstract was available for review; ^#^ Same study covered three countries.

Based on two Asian studies, one third [42] and one half [45] of subjects smoked more than 20 cigarettes per day, while two other studies reported a daily rate of 10–20 cigarettes [1,43]. Another study in China reported an average intake of 10 cigarettes per day [41]. Based on two studies in Africa, more than half of the physician smoked more than 15 cigarettes per day [28,30]. In most studies in Central and Eastern Europe, physicians smoked between 10 and 20 cigarettes per day. A 2006 study in Ukraine [27] found that 66% of physicians smoked less than a half-pack per day. Central and South American physicians had notably lower rates of smoking: in all but one study, the average numbers of cigarettes smoked daily were below 10. In one Brazilian study [22], the rate was 14 cigarettes per day.

#### 3.2.5. Regional Variations in Smoking

The smoking prevalence rate among physicians in Central and South America ranged from 6.4% [25] to 62% [65] (Median: 25%). The prevalence in Central/Eastern Europe ranged from 14% among Ukrainian physicians [27] to 59% in a 1993 Latvian study [1] (Median: 37%). In Asia the prevalence rate ranged from as low as 9.2% among Lao physicians [31] to as high as 50% among Mongolian military doctors [1] (Median: 17.5%). Prevalence rates among African physicians who smoked ranged from 3% at two Nigerian teaching hospitals [16] to 40% in both Egypt and Algeria during a multi-country survey [11] (Median: 29%). Again, the lack of continuity in methods and the broad timeframe makes regional comparisons tenuous. Additionally, prevalence of smoking by physicians may differ heavily between regions of the same country, so single data points may sharply under or over-estimate country prevalence [25].

#### 3.2.6. Smoking in Front of Patients

The proportion of physicians who smoked in front of patients or at the clinic/hospitals was as high as 70% among physicians in Senegal [28], 66% in Costa Rica [63], and 50% among Egyptian physicians [15]. In a study by Jiang *et al.* [40], one third of Chinese physicians smoked in front of patients and almost all smoked during their work shift. A few studies showed physicians with more restraint, with a majority of smoker physicians in some regions avoiding smoking in front of patients or banning it in the workplace [29,55,64]. 

### 3.3. Quitting Smoking among Physicians

Several studies reported quit rates, which were mainly natural quit rates (*i.e.*, cold turkey) among physicians (*i.e.*, former smoking, defined as smoking in the past but not at the time of survey). Twenty-five studies reported the prevalence of former smokers across studies, as shown in Table 2. Former smoking rates ranged from as low as 2% among Chinese physicians [41] to as high as 40% among Costa Rican physicians [63].

Health was the most common reason given by physicians for quitting smoking, which sometimes included concern for the health of others as well as for their own health [20,44,48,61]. Heavy smokers had a more difficult time quitting and were more resistant to accepting quitting support [27]. One study in Armenia indicated that female smokers were 1.5 times more likely to indicate a desire to quit than male smokers [46].

**Table 2:** Former smoking rates among physicians as reported by 25 studies.

Author	Country	Year	Former Smokers
Perrin, *et al.* [46]	Armenia	2006	10%
Squier, *et al.* [27]	Ukraine	2006	22%
Parna, *et al.* [48]	Estonia	2005	32% (males)
Hodgetts, *et al.* [8]	Bosnia and Herzegovina	2004	17%
Akvardar, *et al.* [50]	Turkey	2004	13%
Innos, *et al.* [17]	Estonia	1982	13%
Vanphanom, *et al.* [31]	Laos	2011	18.4%
Zhou, *et. al.* [6]	China	2010	5%
Shi, *et al.* [34]	China	2010	10.1%
Fadhil, *et al.* [36]	Bahrain	2009	10%
Merill *et al.* [26]	Jordan	2008	17%
Yan, J., *et al.* [38]	China	2007	6.3%
Jiang, *et al.* [40]	China	2007	3%
Smith, *et al.* [41]	China	2006	2% (males)
Mohan, *et al.* [20]	India	2006	38% (males)
Yaacob and Abdullah [43]	Malaysia	1993	13%
Supramaniam [45]	Malaysia	1980	10%
Rady and Sabbour [15]	Egypt	1997	12% (males)
Callander and Rocke [29]	South Africa	1986	23%
Ballal [30]	Sudan	1984	13% (males)
Mirra and Rosemberg [25]	Brazil	2007	34%
Viegas, *et al.* [21]	Brazil	2006	23%
Barnoya and Glantz [61]	Guatemala	2002	35%
Grossman, *et al.* [63]	Costa Rica	1999	40%
Sansores, *et al.* [19]	Mexico	1999	20%

#### Intention to Quit and Past Quitting Attempt

Twenty-two articles reported on desire to quit or previous quit attempts among physicians who smoked. As many as 60% of Indian physicians who smoked had tried to quit in the past year [20]; one-third of Chinese physicians who smoked wanted to quit [42] and 80% of South African physicians thought of quitting at some point in the future [29]. In Central and Eastern Europe, between 50% [48] and 62% [56] of smoking physicians had tried to quit, and 68–75% expressed an intention to quit smoking [52,55]. In Latin America, 73% of physicians who smoked had tried to quit in the past [19,22] and 76% expressed an intention to quit [61].

### 3.4. Smoking Cessation Counseling Practices among Physicians

In most studies where the question was raised, physicians and other health care workers felt that helping patients to quit was part of their job [13,29,47]. In Bosnia-Herzegovina [8] and Senegal [16], physicians felt that patients were likely to listen to their smoking cessation advice. However, in Turkey [56] and China [42], the physicians saw their role as minor in the patient’s decision to quit. 

#### 3.4.1. Asking about Tobacco Use

In seven studies, at least three-quarters of physicians, more frequently among non-smoker physicians, reported that they questioned their patients about tobacco use or knew their smoking status [16,27,29,47,61,63]. In a Turkish study [49], physicians asked about smoking almost half the time, while only 30% of Cuban physicians always asked about smoking [60]. 

#### 3.4.2. Advising Patients to Quit Smoking

The prevalence of advice to quit differed across studies. In Estonia, nearly all physicians (96%) reported advising patients to quit [56]. In China, 70% of physicians had counseled patients to quit within the last year [42]. About half the physicians in Malaysia [45], Guatemala [61], and Russia [13] advised patients to quit, while less than one third of physicians in a Chinese study [6] did so. In South Africa [29] and Malaysia [45], advice to quit depended upon the patient having a condition associated with smoking. The most commonly used methods to encourage patients to quit were brief counseling and education about the dangers of smoking in several studies [6,27,45,63]. 

#### 3.4.3. Provision of Counseling/Pharmacological Therapy and Follow-up Arrangements

Giving cessation counseling and making follow-up arrangements were not common across studies. For example, less than one-quarter of Ukrainian physicians provided advice on quitting and just 10% provided nicotine replacement therapy (NRT) or referrals to quitting clinics [27]. In a Turkish study, less than 5% of physicians always arranged follow-up visits or assisted patients with a cessation plan [49]. Only 1% of Nigerian physicians reportedly had prescribed pharmacotherapy for smoking cessation [16]. Providing behavioral counseling or prescribing NRT was also not common among Costa Rican [63], Russian [13], and Guatemalan physicians [61]. Two studies showed more promising results: in Estonia, about two-thirds of surveyed physicians spent time counseling smokers [56]; and in Brazil, slightly more than half of the surveyed physicians provided either a prescription or a referral along with advice to quit [22]. Factors that influenced physicians’ engagement in smoking cessation included physicians’ smoking status [40,44,61], their attitudes towards smoking [20,40,44,48,49], and confidence level in their smoking cessation skills [6,20]. 

### 3.5. Interventions to Promote Smoking Cessation among Physicians

Literature describing programs promoting smoking cessation among physicians is scarce in developing countries. Only three studies provided brief information on the nature of such cessation interventions. Mirra and Rosemberg [25] reported that in Brazil there is few tobacco use reduction or smoking cessation programs that targeted physicians, but details on those programs were not available. Barnoya reported that physician smokers who had quit in the past had primarily used the “cold turkey” method, and none had tried NRT [61]. Viegas [21] reported, also in Brazil, that about three-quarters of smoking physicians were advised by their own peer doctors to quit. A few studies suggested different measures to reduce tobacco use among physicians [25,27,29,41,55,63]. These include introducing tobacco control education in the medical school; a policy initiative to ban smoking in health facilities; training physicians on smoking cessation as part of national action plan; an awareness campaign customized to physicians; and quitting support services customized for physicians. 

## 4. Discussion and Conclusions

The study summarizes studies from across a number of developing countries on tobacco smoking and smoking cessation practices among the physicians. We found a substantial and informative body of literature on smoking among physicians in developing countries. A higher number of studies were identified from Central/Eastern Europe and Central/South America than from Asia and Africa. A significant number of studies were conducted starting in 2000, which might be due to the aggressive tobacco control activities that were initiated by the WHO and other advocacy groups. We found that the focused region or country to study tobacco use among physicians varied between the studies conducted during 1980s and those conducted in the last decade. For example, no study among physicians was reported in China during 1980s while from 1999 onwards nine studies were reported. This likely reflects the fact that smoking was so socially acceptable in China a decade ago that even the public health workforce did not feel the need to conduct surveys of physicians smoking behavior and cessation practices. However, regardless of the origin or period of the study, tobacco use among physicians in many developing countries remains a major public health concern. 

We found variation in smoking rates across the studies that are related to several factors including methodological differences in data collection, differing sample sizes across studies, differing time periods, and variations in the smoking culture across the countries. Overall, our findings strongly suggest that the prevalence of smoking among physicians in developing countries is high. This high prevalence of smoking among physicians has implications for the general population because continued role modeling of smoking by physicians undermines the messages that smoking is harmful and that quitting is important [4]. Studies find that non-smoking physicians are more successful in getting their patients to attempt to quit than smoking physicians [67]. Moreover, physicians who smoke may increase public skepticism about quitting, with people inclined to ask why they should stop smoking when their doctor continues to smoke [68]. 

Many smoker physicians in this review had tried to quit; many were successful in their past quitting efforts, while some were unsuccessful. Identification of factors in well-designed studies that were associated with success and failure could guide policy makers in designing appropriate interventions. Many physicians initiated smoking while they were at medical schools [20,40,46]; therefore, there is a need to design interventions that would discourage tobacco use initiation at the course of their studies at medical schools. In most of the studies, male physicians smoked more than their female counterparts. The difference was greater in Asian studies, suggesting cultural sensitivity in approving smoking by professional women in Asian countries. Although the regional differences may reflect cultural differences regarding smoking by women, continuous efforts should be maintained to keep the prevalence of female smokers low in these countries in response to aggressive promotion of tobacco use among Asian women by the tobacco industry [69]. At the same time, there is a need for targeted programs to promote cessation among male physicians who smoke [6]. Although ex-smokers are few in number, they can serve as role models in encouraging quitting and can provide social support to physicians who want to quit [7,33]. Other innovative approaches such as mobilizing key leaders to promote smoking cessation could also play a major role in encouraging physicians to quit smoking [7].

Smoking in front of patients or at worksites was prevalent in all the regions. This practice might be related to the lack of awareness about their professional role and reinforcement of smoke-free hospital policies. It might also relate to the doctors’ physical dependency on nicotine, myths about smoking that tobacco use represent high social status and inappropriate attitudes towards smoking cessation [7,70]. Strengthening the implementation of smoke-free hospital policies in combination with providing smoking cessation training and support for health care workers who smoke could reduce smoking within hospitals. 

The review found that many physicians, regardless of their smoking status, are missing opportunities to advise or counsel patients on quitting smoking. In a study by Zou *et al.* [6], more positive beliefs and higher confidence level were associated with asking and advising patients to quit. This indicates the need for professional training to address local doctors’ beliefs, attitudes and confidence levels in relation to smoking cessation. The review also revealed that only a minority of physicians prescribed medications such as NRT, which provide effective pharmacological support for smoking cessation. The low use of pharmacotherapy might be explained by doctors’ doubts about the effectiveness of such products [6,7] or the products’ unavailability in the relevant country, or financial barriers in buying NRT.

Although reducing smoking among physicians and involving them in tobacco control activities is critical in combating tobacco-induced morbidity and mortality in these developing regions of the world, this review suggests that the evidence-based smoking cessation programs targeting physicians are scarce. This reflects the lack of interest among policy makers and researchers to focus on reduction of tobacco use among physicians. Evidence from both developing [27,48] and developed [71] nations showed that the rate of asking about smoking and advising to quit smoking was uncommon among smoking physicians. Therefore, organized programs to address tobacco use among physicians should be a priority for developing countries, which would have an impact on the smoking rate at the population level. As other allied health professionals (*i.e.*, nurses, physiotherapist, and community health workers) also play a critical role in the healthcare delivery in developing countries and have frequent contacts with the public, engaging the allied health professionals in the tobacco use reduction and cessation effort would be useful. For example, community health workers played a key role in the delivery of other preventive health programs (*i.e.*, immunization, breastfeeding) and treatment for malaria in low income countries, engaging them in the tobacco control effort would have great population health impact.

As evidence-based smoking cessation interventions are already available in developed countries, testing and adopting them in developing country settings should be a priority. Involvement of professional societies, medical associations, tobacco-control advocates and other influential local leaders and hospital executives, non-governmental organizations, and international agencies is necessary to reach physicians with cessation interventions and encourage them to act as tobacco-control advocates. Intervention programs should address influencing factors such as physician characteristics (e.g., smoking status, perception of self as role model), structural factors (e.g., time, and reimbursement), and cessation-specific knowledge and skills. As social context [72] and myths [73] may play roles in sustaining smoking behavior and quitting effort, future intervention programs should also address these issues. The WHO FCTC has dramatically altered the global tobacco control environment and public health professionals should utilize this opportunity to engage physicians in the tobacco control effort and address tobacco use by the physicians. Global tobacco control efforts should highlight tobacco use by the physicians as one of the major priorities and work with professional societies and government bodies to address the issue in an organized manner. Regular data collection from physicians using standard questionnaires, such as Global Health Professionals Survey [74], would be useful to monitor the tobacco use trends over time and assess the impact of any tobacco control policy or targeted interventions among physicians. 

The review has both strengths and limitations. To the best of our knowledge, it is the first comprehensive review on the topic to focus on developing countries. Methodological differences across studies (*i.e.*, sample sizes, how the data were collected, gender ratio of the collected subjects, the definition used to define current smokers, and the date in which the studies were conducted) were unavoidable limitations, as was our access to published data only. Finally, the findings we summarized are based on the reports in the published papers. No attempts were made to validate the findings or conclusions of the reported studies. Therefore, these limitations should be considered when extrapolating the findings of the current paper. Also, this review focused mainly on cigarette smoking as most of the available reports was based on cigarette smoking among the physicians. However, this should be noted that tobacco use encompasses more than cigarette smoking. Future studies should consider physician’s use of and counseling behaviors regarding other forms of tobacco (smoked and smokeless) use.

Overall, this review suggests that while smoking habits of physicians vary across different developing regions; prevalence rates tend to be higher than among physicians in developed countries. Also, quitting rates were low among the physicians, and the delivery of advice on quitting smoking was not common across the studies. Although the number of studies from each of the global regions under consideration was unbalanced in the review, this may reflect patterns of tobacco control activities targeting physicians in each region. Future review might focus on a single geographic region and explore studies by cultural norms to document culturally sensitive factors associated with physician smoking or delivery of cessation intervention. Future research would benefit from the exploration of the factors discussed in this review to determine the facilitating factors to reduce tobacco smoke among physicians and to promote physician-led smoking cessation and tobacco use reduction intervention. 
